# Abnormal anther development leads to lower spikelet fertility in rice (*Oryza sativa* L.) under high temperature during the panicle initiation stage

**DOI:** 10.1186/s12870-021-03209-w

**Published:** 2021-09-20

**Authors:** Qiuqian Hu, Wencheng Wang, Qifan Lu, Jianliang Huang, Shaobing Peng, Kehui Cui

**Affiliations:** National Key Laboratory of Crop Genetic Improvement, Ministry of Agriculture Key Laboratory of Crop Ecophysiology and Farming System in the Middle Reaches of the Yangtze River, College of Plant Science and Technology, Huazhong Agricultural University, Wuhan, China

**Keywords:** Anther dehiscence rate, Disturbed anther walls, Grain yield, Heat tolerance, Pollen viability, Rice (*Oryza sativa* L.), Tapetum

## Abstract

**Background:**

Decreased spikelet fertility is often responsible for reduction in grain yield in rice (*Oryza sativa* L.). In this study, two varieties with different levels of heat tolerance, Liangyoupeijiu (LYPJ, heat susceptible) and Shanyou63 (SY63, heat tolerant) were subjected to two temperature treatments for 28 days during the panicle initiation stage in temperature/relative humidity-controlled greenhouses: high temperature (HT; 37/27 °C; day/night) and control temperature (CK; 31/27 °C; day/night) to investigate changes in anther development under HT during panicle initiation and their relationship with spikelet fertility.

**Results:**

HT significantly decreased the grain yield of LYPJ by decreasing the number of spikelets per panicle and seed setting percentage. In addition, HT produced minor adverse effects in SY63. The decreased spikelet fertility was primarily attributed to decreased pollen viability and anther dehiscence, as well as poor pollen shedding of the anthers of LYPJ under HT. HT resulted in abnormal anther development (fewer vacuolated microspores, un-degraded tapetum, unevenly distributed Ubisch bodies) and malformation of pollen (obscure outline of the pollen exine with a collapsed bacula, disordered tectum, and no nexine of the pollen walls, uneven sporopollenin deposition on the surface of pollen grains) in LYPJ, which may have lowered pollen viability. Additionally, HT produced a compact knitted anther cuticle structure of the epidermis, an un-degraded septum, a thickened anther wall, unevenly distributed Ubisch bodies, and inhibition of the confluent locule, and these malformed structures may be partially responsible for the decreased anther dehiscence rate and reduced pollen shedding of the anthers in LYPJ. In contrast, the anther wall and pollen development of SY63 were not substantially changed under HT.

**Conclusions:**

Our results suggest that disturbed anther walls and pollen development are responsible for the reduced spikelet fertility and grain yield of the tested heat susceptible variety, and noninvasive anthers and pollen formation in response to HT were associated with improved heat tolerance.

**Supplementary Information:**

The online version contains supplementary material available at 10.1186/s12870-021-03209-w.

## Background

Plant responses to temperature stress are receiving increased attention due to growing awareness about global warming. High temperature (HT) stress at different periods of reproductive growth has detrimental effects on rice yield, mainly via decreasing pollen viability, spikelet fertility and the grain filling percentage [1–4]. Importantly, almost all spikelets were sterile when high temperature stress occurred at the early microspore stage after meiosis [5]. With temperature increases induced by global warming in the future, developing panicles will experience HT stress more frequently. Therefore, it is important that measures are taken to study the mechanism of HT injury on rice panicle development and implement reasonable procedures to alleviate high temperature injury during panicle initiation.

Abnormal panicle development is one of the main factors limiting grain yield formation under HT stress. At panicle initiation, HT resulted in attenuated differentiation of secondary branches and an increased number of degraded branches, and fewer spikelets per panicle, as well as reduced grain length, grain width, grain area, and grain weight [3]. Decreased pollen viability are tightly associated with abnormal anther development [6–9] and responsible for spikelet sterility under HT during panicle initiation [3]. Reduced spikelet fertility under HT at the flowering stage was attributed to abnormalities in anther dehiscence [10], pollen shedding and pollen germination [4, 11]. Pollen production, the amount of pollen grains on the stigma, in vivo pollen germination on the stigma, and hidden stigmas were positively associated with spikelet fertility under HT at the flowering stage [1, 2, 12–14]. These previous reports show that the normal development of reproductive organs is a necessary perquisite for spikelet fertility and grain yield formation under heat stress.

Anther development plays an important role in pollen viability and spikelet fertility; however, anther development is frequently affected by HT stress [2, 15, 16]. Anther development can be divided into 14 stages according to the cellular features of anthers [17], and the meiosis stage and microspore development stage were found to be sensitive to HT stress in rice [5, 6, 18]. High temperature resulted in disordered pollen development in wheat, sorghum, tomato and maize [7, 19–21], which led to ovoid pollen grains with dehydrated exine [20], shriveled and collapsed pollen grains with rough exine walls, and collapsed germinal apertures [21]. Deng et al. [6] found that tetrad cells failed to separate under HT during the meiosis stage, while meiocytes adhered to each other. However, the process of pollen development and its relationship with spikelet fertility and yield in rice exposed to HT stress during panicle initiation are not well understood.

The anther wall consists of four layers: epidermis, endothecium, middle layer, and tapetum. At the tetrads stage (stage 8 of anther development), the middle layer disappears and the tapetum is degraded. At the vacuolated microspore stage (stage 10 of anther development), the tapetum thins and microspores become vacuolated and adhere tightly to the tapetum. At the pollen maturity stage (stage 13 of anther development), only the epidermis and endothecium remain, pollen grain filling is completed, and the stomium is split for dehiscence [8]. HT stress often disturbs anther development [6, 8, 22, 23]. The development of the endothecium, epidermis, and stomium at stage 13 in *Lycopersicon esculentum* Mill were disrupted under HT [22], and cells of the epidermis in rice mature anthers were arranged loosely when subjected to high temperature at the heading stage [23].

Normal and timely degeneration of the tapetum is essential for microspore development, pollen adhesion and germination on the stigma, and pollen viability [5, 8, 9, 24]. The reported effects of HT on the initiation of tapetum degeneration have not been consistent in previous studies. Suzuki et al. [9] observed advanced tapetum degeneration and pollen sterility in snap bean plants exposed to HT conditions; however, HT delayed tapetum degeneration in cotton [25] and rice [6], resulting in low pollen viability. In addition, Endo et al. [5] observed that 3 days of HT treatment during meiosis did not affect the tapetum degradation process in rice, but pollen viability was decreased by this treatment. These data show the inconsistency of reported tapetum degeneration responses to HT conditions and demonstrate that the effect of HT on tapetum degeneration requires further study.

Anther dehiscence is essential for pollen spreading and pollination, which are tightly associated with spikelet fertility [10, 11]. The anther dehiscence percentage in rice decreased under HT during flowering [10, 11]. There are several cell layers of anther wall between the locule and the lacuna (the space between the septum adhering to the anther wall and the stomium). Thickened cell layers and delayed locule opening in response to HT, leading to decreased spikelet fertility [26]. However, Bagha [27] found that the cell layers were not affected by HT, and failure in lysis of the septum cell wall prevented anther locules from opening during panicle initiation in rice. Therefore, probing the relationship between anther dehiscence and anatomic structures is an important step toward a deeper understanding of the effects of heat stress on spikelet fertility.

In a previous study, we found that rice cultivar Liangyoupeijiu (LYPJ) had low spikelet fertility and was susceptible to HT during panicle initiation, whereas Shanyou63 (SY63) was more tolerant to HT with high spikelet fertility [3]. However, the underlying mechanism for the different responses of spikelet fertility to HT treatment in the two cultivars is not elucidated yet. Therefore, the objective of this study was to (1) illustrate changes in anther development under HT during panicle initiation and their relationship with spikelet fertility; (2) further compare the varietal difference in response of anther development to heat stress.

## Results

### Effects of growth temperature on rice yield formation

HT treatment significantly decreased the grain yield, seed setting percentage, and spikelets per panicle of LYPJ, but it had no significant effects on the grain yield or yield components of SY63 (Table 1). The grain yield of LYPJ was reduced by 84% under HT when compared with that under CK, and this change was accompanied by reductions in spikelets per panicle (35%), seed setting percentage (69%), and 1000-grain weight (17%). The change in the grain yield of SY63 (18%) was smaller than that of LYPJ due to small reductions in 1000-grain weight (7% in SY63) and grain setting percentage (20% in SY63). HT had no significant effects on the number of panicles per plant or biomass of either cultivar (Table 1).

**Table 1:** Effects of high temperature treatment on yield and yield components in LYPJ and SY63

Cultivars	Treatment	Yield	Panicles	Spikelets	Seed setting percentage	1000-grain weight	Biomass
		g plant^−1^	No. plant^− 1^	No. panicle^− 1^	%	g	g plant^− 1^
LYPJ	CK	20.7 ± 1.4 ^a^	11.3 ± 0.3 ^a^	153.2 ± 5.2 ^a^	53.3 ± 0.8 ^a^	22.3 ± 0.6 ^a^	87.0 ± 1.3 ^a *^
	HT	3.3 ± 0.8 ^b^	11.0 ± 0.0 ^a^	100.2 ± 6.5 ^b^	16.4 ± 4.5 ^b^	18.5 ± 0.1 ^b^	81.4 ± 0.7 ^a^
SY63	CK	25.5 ± 0.8 ^a^	11.3 ± 0.3 ^a^	115.3 ± 5.7 ^a^	81.1 ± 1.0 ^a *^	24.2 ± 0.5 ^a *^	75.3 ± 3.4 ^a^
	HT	21.0 ± 1.4 ^a*^	11.0 ± 0.6 ^a^	130.9 ± 2.2 ^a^	65.1 ± 4.7 ^a*^	22.5 ± 0.5 ^a*^	69.7 ± 3.2 ^a^

*CK* control temperature, *HT* high temperature. Data are the average of three replicates ± standard error (*n* = 3). The different superscript lower-case letters indicate significance between the temperature treatments for the same cultivar at *P* < 0.05. Asterisk indicates significance between the two cultivars for same temperature treatment at *P* < 0.05

### Effects of HT on fertility and anther dehiscence

**Fig. 1:**
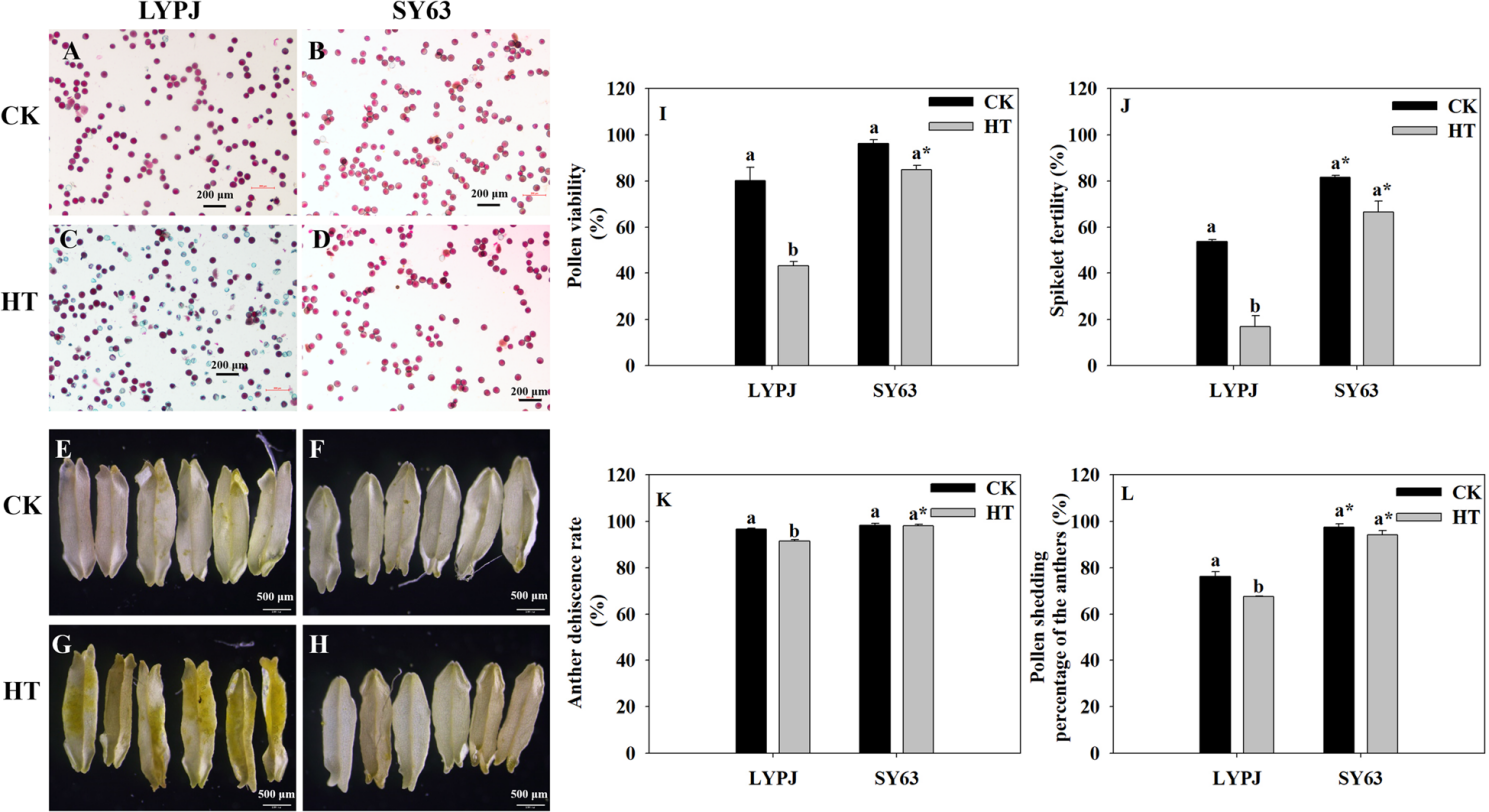
Effects of high temperature treatment on fertility, anther dehiscence and pollen shedding in LYPJ and SY63. **A**, **B**, **C**, and **D** show pollen viability. The reddish-purple pollen grains are fertile, and the green pollen grains are sterile (scale bar = 200 μm); **E**, **F**, **G**, and **H** show dehisced anthers at flowering using a stereomicroscope (scale bar = 500 μm); **I**, pollen fertility; **J**, spikelet fertility; **K**, anther dehiscence rate; **L**, pollen shedding percentage of the anthers. CK, control temperature; HT, high temperature. Data are the average over three replicates ± standard error of the mean (*n* = 3). The different letters indicate significance between temperature treatments for the same cultivars at *P* < 0.05. Asterisk indicates significance between the two cultivars for same temperature treatment at *P* < 0.05

More non-viable pollen grains (colored in green, Fig. 1C) and anthers with many pollen grains (colored in yellow, Fig. 1G) were observed in LYPJ under HT than under CK (Fig. 1A and E); however, no obvious difference in the numbers of green pollen grains or yellow anthers were found between CK and HT in SY63 (Fig. 1B, D, F and H). Accordingly, HT treatment markedly decreased pollen viability by 46% (Fig. 1I) and spikelet fertility by 69% (Fig. 1J) in LYPJ, but no significant reduction was observed in SY63 under CK and HT treatments. The anther dehiscence rate was decreased by 5% in LYPJ under HT in comparison with those under CK, and the pollen shedding percentage of the anthers was decreased by 11% (Fig. 1K and L), whereas no significant differences was observed in either trait between CK and HT in SY63. Additionally, the absolute values of the pollen viability, spikelet fertility, anther dehiscence rate, and pollen shedding percentage of the anthers of SY63 were 42, 50, 7 and 27 percentage points higher, respectively, than those of LYPJ under HT (Fig. 1I, J, K and L).

### Scanning electron microscopic observations of anther characteristics

**Fig. 2:**
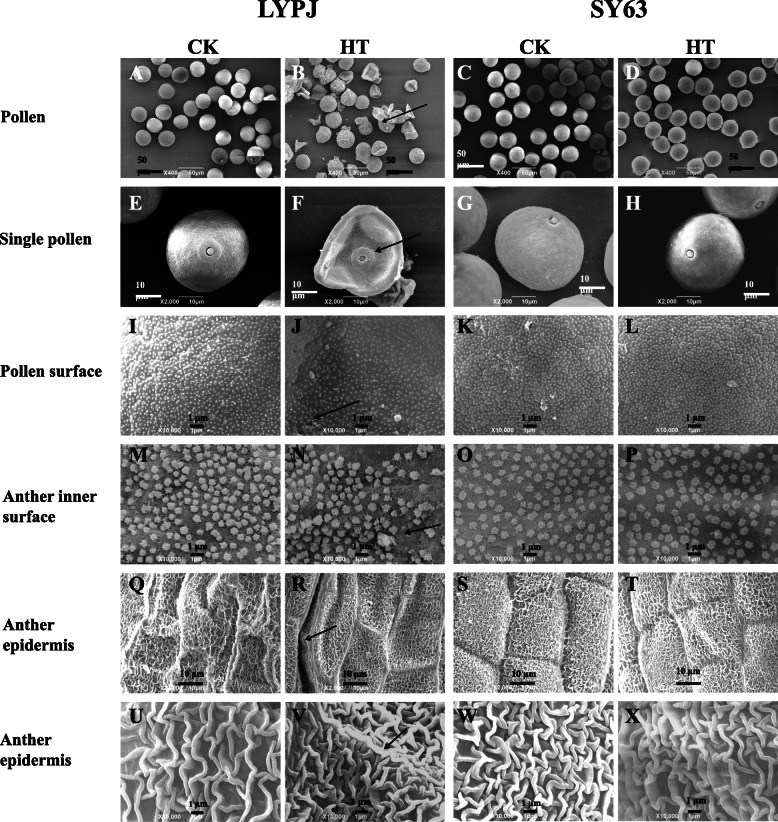
Scanning electron micrographs of mature anthers from LYPJ and SY63 plants. **A**, **B**, **C**, and **D** show scanning electron micrographs of pollen grains (scale bar = 50 μm); **E**, **F**, **G**, and **H** show magnified single pollen grain (scale bar = 10 μm); **I**, **J**, **K**, and **L** show the pollen surface (scale bar = 1 μm); **M**, **N**, **O**, and **P** show the anther inner surface (scale bar = 1 μm); **Q**, **R**, **S**, and **T** show the anther epidermis at 2000x magnification (scale bar = 10 μm); **U**, **V**, **W**, and **X** show the anther epidermis at 10000x magnification (scale bar = 1 μm). The arrows indicate shriveled pollen (**B**), collapsed pollen grain with a hollow germinal aperture (**F**), abnormal deposition of sporopollenin on the pollen surface (**J**), uneven distribution of Ubisch bodies on the anther inner surface (**N**), and compacted anther epidermis (**R**, **V**). CK, control temperature; HT, high temperature

Anther and microspore characteristics were analyzed by scanning electron microscopy. As shown in Fig. 2, almost all pollen grains were round-shaped under CK (Fig. 2A and C), and the pollen surface was smooth (Fig. 2E and G); however, HT treatment resulted in shriveled and collapsed pollen grains with a hollow germinal aperture (Fig. 2B and F), especially in LYPJ. Additionally, typically granular sporopollenins on the pollen surface were observed under CK (Fig. 2I and K); however, sporopollenin deposition was abnormal under HT, especially in LYPJ (Fig. 2J). The Ubisch bodies were evenly distributed and had typically sharp protrusions under CK (Fig. 2M and O); however, HT resulted in unevenly distributed Ubisch bodies with blunt protrusions in LYPJ (Fig. 2N) and had no effect on SY63 (Fig. 2P). The surface of the epidermis showed varietal variation between LYPJ (Fig. 2Q) and SY63 (Fig. 2S). Striate ornamentation of anther epidermis cuticle had a radial and longitudinal pattern for the two cultivars. However, the surface of the epidermis was wrinkled (Fig. 2Q, R) in LYPJ and smooth in SY63 (Fig. 2S, T) regardless of temperature treatments. HT treatment resulted in a compact knitted anther cuticle structure on the epidermis of LYPJ (Fig. 2R, V) in comparison with CK (Fig. 2Q and U). Generally, HT treatment had no substantial effect on epidermis characteristics (Fig. 2T and X) in comparison with those of plants exposed to CK (Fig. 2S and W).

### Transmission electron microscopic observations at stage 10

**Fig. 3:**
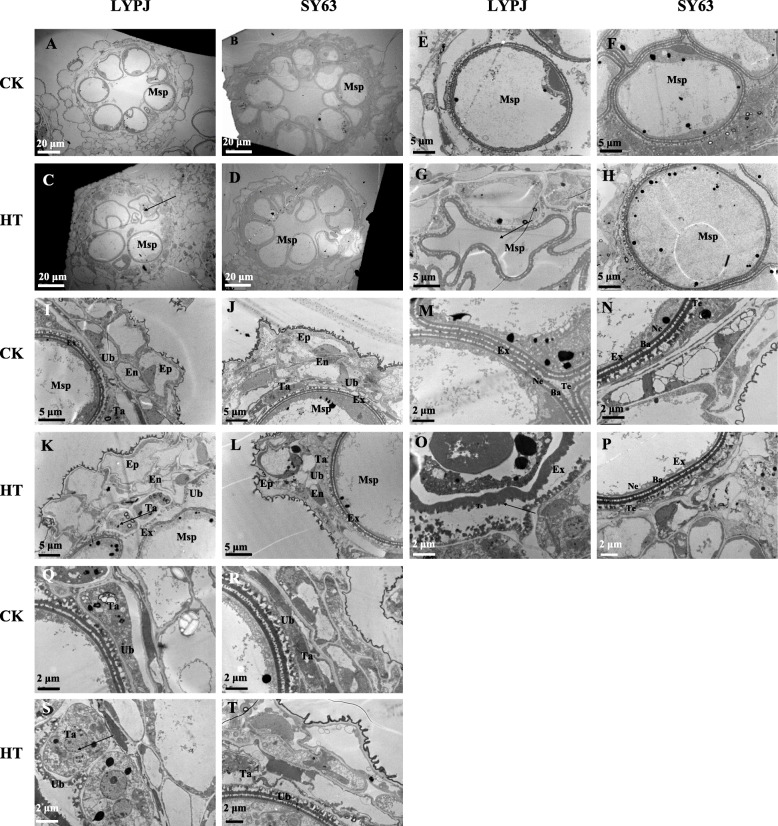
Transmission electron micrographs of anthers at stage 10 in LYPJ and SY63 plants. **A**, **B**, **C**, and **D** show anther locules (scale bar = 20 μm); **E**, **F**, **G**, and **H** show individual microspore (scale bar =5 μm); **I**, **J**, **K**, and **L** show anther walls (scale bar = 5 μm); **M**, **N**, **O**, **P** show pollen exine (scale bar = 2 μm); **Q**, **R**, **S**, **T** show Ubisch bodies and tapetum cells (scale bar = 2 μm). Ep, epidermis; En, endothecium; Msp, microspores; Ta, tapetal layer; Ex, pollen exine; Ne, nexine; Te, tectum; Ba, bacula; Ub, Ubisch bodies. CK, control temperature; HT, high temperature

Anther ultra-structures in stage 10 were subjected to further observation and quantification via transmission electron microscopy (Fig. 3). Spherically vacuolated microspores were closely adhered to the tapetum under the CK conditions for both cultivars (Fig. 3A, B, E, F, I and J). However, HT led to the collapse of microspores in LYPJ, as well as degradation of the cytoplasm and organelles (Fig. 3G). In addition, the tapetum in LYPJ was vacuolated and hypertrophic under HT (Fig. 3C, K and S) in comparison with that under CK (Fig. 3A, I and Q). In comparison with plants under CK (Fig. 3M and Q), LYPJ grown under HT showed unevenly distributed Ubisch bodies (Fig. 3O and S) and an obscure outline of the pollen exine with three malformed layers (collapsed bacula, disordered tectum, and no nexine) (Fig. 3O). Generally, the characteristics of the microspores and anther walls mentioned above were not significantly changed in SY63 under HT (Fig. 3D, H, L, P and T).

Quantitatively, HT treatment significantly increased the average area of tapetum cells by 136% in LYPJ under HT in comparison with CK (Table 2, Fig. 3Q and S), and it had no effect on the tapetum cells of SY63 (Table 2, Fig. 3R and T). The tapetum cell area of LYPJ was twice as that of SY63 under HT, but the tapetum cell areas of LYPJ and SY63s were similar under the CK conditions (Table 2, Fig. 3Q and S). In LYPJ, the number of vacuolated microspores per locule was significantly decreased from 7.2 under the CK treatment to 1.5 under HT (Table 2, Fig. 3A and C), but this number was reduced by only one in SY63 (Table 2, Fig. 3B and D).

**Table 2:** Effects of high temperature treatment on anther characteristics at stage 10 in LYPJ and SY63

Cultivars	Treatment	Average area of tapetum cell	Number of vacuolated microspores	Number of stomium cells	Number of septum cells	
					between bundle sheath and sub-stomial lacuna	between stomium and locule
		μm^2^ cell^−1^	No. locule^−1^	No. locule^−1^	No. locule^− 1^	No. locule^− 1^
LYPJ	CK	40.5 ± 1.7 ^b^	7.2 ± 0.3 ^a^	5.5 ± 0.3 ^a^	2.8 ± 0.2 ^a^	0.9 ± 0.1 ^a^
	HT	95.7 ± 4.1 ^a*^	1.5 ± 0.0 ^b^	4.8 ± 0.2 ^a^	3.0 ± 0.0 ^a^	1.1 ± 0.1 ^a^
SY63	CK	44.4 ± 5.3 ^a^	8.2 ± 0.2 ^a^	5.5 ± 0.3 ^a^	2.3 ± 0.2 ^a^	0.5 ± 0.1 ^a^
	HT	48.5 ± 1.9 ^a^	7.2 ± 0.3 ^a*^	4.3 ± 0.3 ^a^	2.5 ± 0.3 ^a^	0.8 ± 0.0 ^a^

*CK* control temperature, *HT* high temperature; Data are the average over three replicates ± standard error (*n* = 3). The different superscript lower-case letters indicate significance at *P* < 0.05 between the temperature treatments for the same cultivar. Asterisk indicates significance at between the two cultivars for same temperature treatment *P* < 0.05

HT had no effect on the number of stomium cells for either variety (Table 2 and Fig. 5), but HT reduced the size of the sub-stomial lacunas between septum cells and stomium cells in LYPJ. In addition, septum cells were not degraded under HT in LYPJ (Fig. 5A and C), but SY63 had larger sub-stomial lacunas and degraded septum cells (Fig. 5B and D).

HT did not significantly affect the number of septum cells between the bundle sheath and sub-stomial lacuna or the number of septum cells between the stomium and locule (Table 2). SY63 had fewer septum cells between the bundle sheath and sub-stomial lacuna, as well as between the stomium and locule, in comparison with LYPJ (Table 2 and Fig. 5).

### Light microscopic observations of anther characteristics

**Fig. 4:**
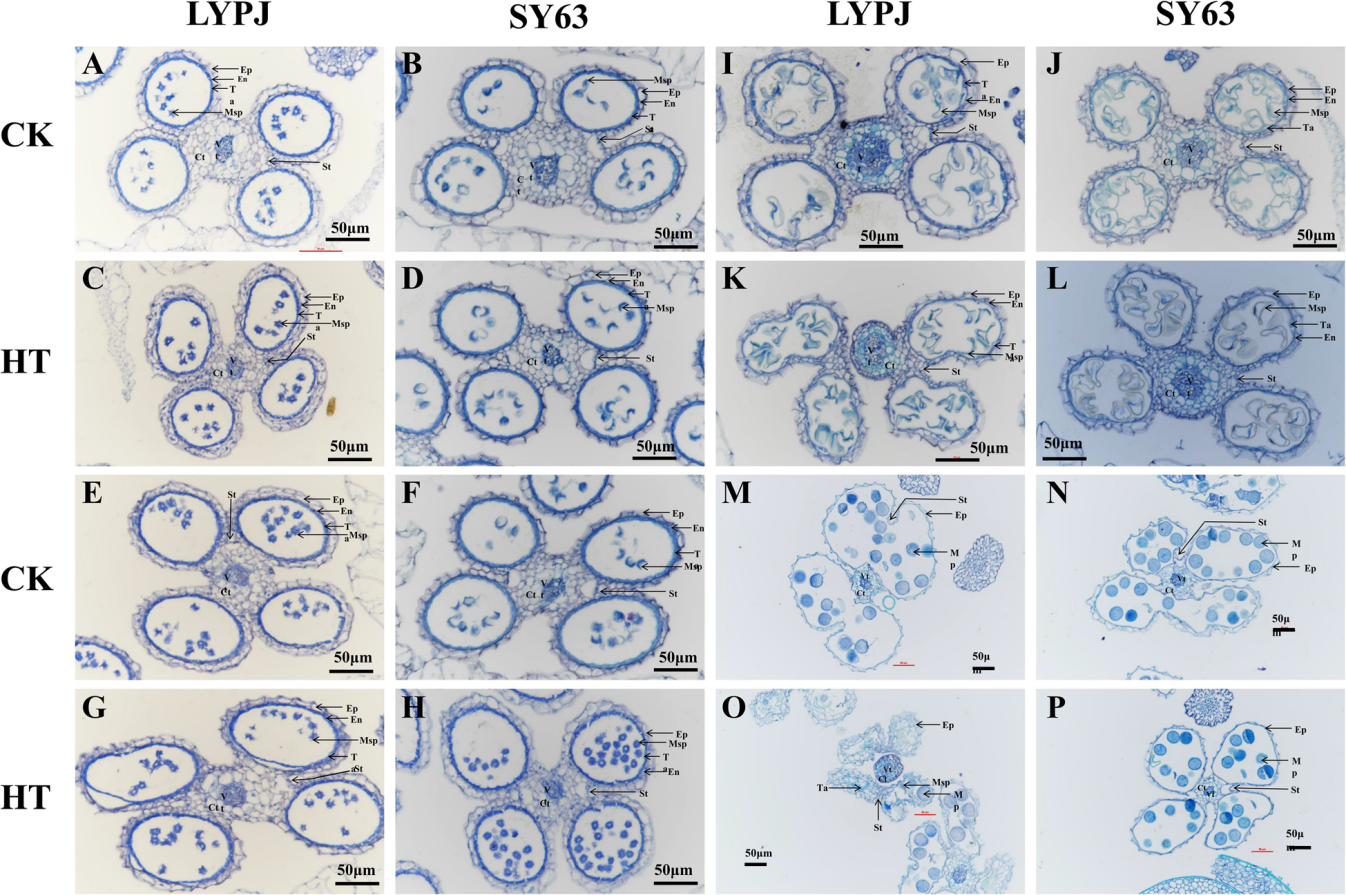
Histological changes of the anthers during anther development in LYPJ and SY63 plants. **A**, **B**, **C**, and **D** show anther cross-sections at stage 8b. **E**, **F**, **G**, and **H** show anther cross-sections at stage 9. **I**, **J**, **K**, and **L** show anther cross-sections at stage 11. **M**, **N**, **O**, and **P** show anther cross-sections at stage 13. Ep, epidermis; En, endothecium; Msp, microspores; Ta, tapetal layer; Mp, mature pollen; Vt, vascular tissue; Ct, connective tissue; St, stomium. Scale bars = 50 μm. CK, control temperature; HT, high temperature

Observations were carried out at 4 stages of anther development via light microscopy. At stage 8b, there were no obvious abnormal changes between the plants exposed to the CK and HT treatments for both varieties (Fig. 4A, B, C and D). At stage 9, when microspores are normally released from the tetrad, the tapetum of LYPJ and SY63 closely adhered to the endothecium under CK conditions (Fig. 4E and F), whereas the tapetum was separated from the endothecium in LYPJ, but not in SY63, under HT (Fig. 4G). At stage 11, condensed and degenerated tapetal cells and vacuolated microspores were observed in LYPJ and SY63 under CK conditions (Fig. 4I and J). In LYPJ under HT, the anther wall layers were disordered and broken, microspores were degraded and appeared irregularly shaped, the tapetum became less condensed, and the lacuna between the septum and stomium became smaller (Fig. 4K), but these structural changes were not observed in SY63 under HT (Fig. 4L). At stage 13 in LYPJ under CK conditions, most of the middle layer and endothecium of the anther wall were degraded, and deeply stained pollen grains and confluent locules were observed (Fig. 4M). However, in LYPJ under HT, the anthers were smaller, the tapetal cells were visible, the anther wall was shrunken and irregularly shaped, and there were fewer fertile pollen grains per anther locule (Fig. 4O and M). However, no significant differences were observed between CK and HT in SY63 (Fig. 4N and P).

## Discussion

### The effects of HT during panicle initiation on yield formation

Most previous studies investigated damage induced by HT during the flowering stage [2, 13, 14, 28–30], while there have been few investigations of the effects of HT during panicle initiation on rice yield formation [2, 3]. Therefore, in this study, we assessed the detrimental effects of HT during panicle initiation in rice.

We found that HT treatment during panicle initiation seriously decreased yield (84%) and yield components of LYPJ (Table 1), with the exception of the number of panicles per plant; however, HT had a smaller effect on yield formation in SY63 in comparison with that observed in LYPJ (Table 1). Moreover, HT slightly increased the number of spikelets in SY63 compared with that of CK (Table 1). Similarly, Wu et al. [3] also observed that HT slightly increased the number of secondary branches per panicle in SY63, and Yang et al. [31] found that daytime warming increased 17 spikelets per panicle in SY63 without significance. Regardless of the observations, it is not sure that the increased temperature is advantageous to panicle formation.

In the study, HT decreased 1000-grain weight by 17% in LYPJ, 7% in SY63, respectively (Table 1). Full spikelet size before heading and grain size at maturity were decreased in both the two cultivars under HT, and the heat sensitive LYPJ had larger reductions in spikelet and grain sizes, especially in length (Table S1). Similar observation was also reported by Wu et al. [3]. Therefore, the lower 1000-grain weight was mainly ascribed to the limitation of spikelet size in two varieties under HT.

In the HT-susceptible genotype LYPJ, the seed setting percentage showed the largest decline among yield components under HT, indicating that the seed setting percentage was more vulnerable to HT in comparison with the other components, which was in agreement with previous reports [3, 31]. In the present study, the heat-tolerant variety SY63 had less yield damage in response to HT, which may be attributed to the high, stable seed setting percentage of this genotype under HT (reduced by 20% in SY63 and 69% in LYPJ). It is important to note that the LYPJ had a relatively low seed setting percentage under CK (Table 1), which may have been due to the natural high temperature (maximum temperature of more than 35 °C for five consecutive days) during early flowering (data not shown). It has been reported previously that the LYPJ genotype is susceptible to heat during flowering [13].

In the present study, LYPJ had very low spikelet fertility under HT (Fig. 1J), and we also found very few half-grains with filling initiation (0.5% in LYPJ and 1.4% in SY63). These findings showed that it was spikelet fertility, rather than the grain filling process, that was responsible for the reductions in seed setting percentage under HT during panicle initiation. These results are similar to those reported in a recent study by Cheabu et al. [32], in which spikelet fertility was the major restriction for reductions of grain yield under HT from booting to maturity. The intrinsic factors responsible for low spikelet fertility under HT include low pollen productivity, low pollen viability, poor anther dehiscence and pollen reception, and poor pollen germination [1, 2, 26]. In this study, HT during panicle initiation significantly decreased the anther dehiscence rate (Fig. 1K). This finding is in accordance with reports by Jagadish et al. [29] and Kobayashi et al. [10], in which rice plants were exposed to HT during the flowering stage. However, Endo et al. [5] and Wu et al. [3] reported that the anther dehiscence rate was not substantially influenced by heat stress during panicle initiation. Moreover, HT treatment was performed for 15 days with a mean daytime maximum temperature of 36.1 °C by Wu et al. [3], whereas HT treatment was performed for 3 days with a mean daytime maximum temperature of 39 °C by Endo et al. [5]. Therefore, the response of the anther dehiscence rate to HT depends on the developmental stage during which plants are exposed to HT and the intensity of the HT treatment. Significant decreases in pollen viability (reduced by 46%), the pollen shedding percentage of the anthers (reduced by 11%) and the anther dehiscence rate (reduced by 5%) were observed in LYPJ plants under HT treatment (Fig. 1C, G, I, K and L), indicating that pollen viability was more vulnerable to HT in comparison with the other components (Fig. 1I, K and L). Therefore, we considered that it was altered pollen viability, rather than changes in pollen shedding or anther dehiscence, that was mainly responsible for the lower spikelet fertility of HT-susceptible cultivar LYPJ under HT during panicle initiation (Fig. 1I, J and K). However, subjecting SY63 plants to HT treatment had no significant effect on any of these traits (Fig. 1). In comparison with LYPJ, the process from anther dehiscence to complete dispersal of pollen grains from the anthers was relatively rapid in SY63 under both tested temperatures (9.0 min under CK and 16.2 min under HT for LYPJ, 2.2 min and 2.5 min for SY63, data not shown). Recently, Wu et al. [13] found that enclosed stigmas of SY63 plants contributed to high spikelet fertility under HT. Therefore, higher spikelet fertility (67%) of HT-tolerant SY63 plants under HT during panicle initiation may be attributed to higher pollen viability (85%), a higher anther dehiscence rate (98%), a shorter period of time required for complete dispersal of pollen grains from anthers, better pollen shedding from anthers (94%), and enclosed stigmas.

### The relationship of pollen viability with anther characteristics

Pollen sterility caused by heat stress has been associated with abnormal anther development in sorghum [19], wheat [20], tomato [22], cotton [25], dwarf bean [9], and rice [2, 33]. In our study, HT treatment disrupted the morphologic structures of the anther wall and spherical microspores in LYPJ (Figs. 2, 3 and 4). Specifically, HT resulted in malformation of the pollen structure (obscure outline of the pollen exine, collapsed bacula, disordered tectum, and no nexine) in LYPJ (Fig. 3G and O) at stage 10. Additionally, we observed aborted pollen grains at stage 13 in LYPJ under HT (Fig. 4O), manifesting as a shriveled and collapsed pollen surface with a hollow germinal aperture and uneven sporopollenin deposition. Previous studies found that the pollen surface severely shriveled under HT in sorghum and maize, and this change was accompanied by poor pollen viability [19, 21]. In the present study, HT did not obviously alter anther development or anther structure in the heat-tolerant variety SY63 (Figs. 2, 3 and 4). These data indicate that abnormal pollen formation was responsible for low pollen viability under HT during panicle initiation, and the heat tolerance of SY63 may be attributed to normal anther development (Fig. 6). In addition, it is worth noting that three topmost spikelets of panicles were selected for anther observation. As we well know, anthers on a panicle might cover several developmental stages in our study (5 stages, Figure S1) and previous reports (4 stages) [34], and the topmost spikelets often develop early. Therefore, it is necessary to further investigate the developmental responses of anthers in spikelets on different positions of panicle.

Moreover, we found that Ubisch bodies had blunt protrusions and an uneven distribution on the inner surface of the anther wall in LYPJ plants under HT (Figs. 2N and 3O). HT also resulted in tight wrinkles of the knitted anther cuticle on the epidermis of LYPJ plants (Fig. 2V). Similarly, Uzair et al. [35] found marginal differences in the patterning of nano-ridges on the outer surface, as well as in the distribution of Ubisch bodies, on the inner surface of anthers in rice *ptc2* mutants, which resulted in decreased pollen viability in comparison with that of wild-type plants. However, HT had no substantial effect on Ubisch bodies or anther cuticles in SY63 (Fig. 2). Ubisch bodies carry a sporophytically produced structural protein that is essential for pollen development [36]. The cuticle on the outer surface of the anther serves as a barrier and protects the microspore/pollen grain from various environmental stresses [37]. These data suggest that well-developed Ubisch bodies and cuticles contribute to the heat tolerance of SY63 under HT during panicle initiation, whereas alterations in Ubisch bodies and cuticle formation may result in pollen sterility in LYPJ (Fig. 6).

In this study, the single-cell tapetum area of LYPJ was larger at stage 10 under HT in comparison with that observed under CK conditions. In LYPJ plants exposed to HT treatment, the tapetum was still observed at stage 13 due to slow degradation, but the tapetum had completely disappeared at this stage in LYPJ plants under CK and in SY63 under HT and CK conditions (Fig. 4). These results show that HT disrupted tapetum degradation in LYPJ. Similarly, halted and incomplete tapetum degradation was reported in rice *ptc2* mutants [35] and rice plants under chilling [38], and as well in tomato and cotton plants under heat stress [25, 39]. Regarding causes for abnormal tapetum degradation, Mamun et al. [38] revealed that vacuolation and hypertrophy of the tapetum under chilling was caused by osmotic imbalance, which was triggered by the reabsorption of callose breakdown products in the absence of OsMST8 activity. Min et al. [25] found that delayed programmed cell death of the tapetum was mainly due to inactivation of starch synthase in cotton under HT. These findings suggest that different regulatory mechanisms govern tapetum degradation in different organisms; however, the mechanism underlying tapetum degeneration retardation in rice under HT is not yet clear.

The tapetum, the innermost layer in the anther wall, serves as an active nutrient source for neighboring microspores [35, 39, 40], and abnormal tapetum degeneration results in pollen sterility in photoperiod and thermosensitive genic male-sterile rice [41, 42] and rice mutants [35]. These previous reports indicate that termination of secretory-type tapetum development and disruption of tapetal functions is partly responsible for pollen viability. In the present study, tapetum degradation in LYPJ was inhibited by HT treatment, whereas SY63 showed nearly tapetum degradation (Fig. 3R and T). Additionally, we observed several differences in the characteristics of the anther walls of LYPJ and SY63 plants following HT treatment. For example, the tapetum did not adhere to the endodermis (Fig. 4G) at stage 9 in LYPJ, at which point tapetum degradation was initiated. In addition, LYPJ had a tightly knitted anther cuticle on the epidermis (Fig. 2R and V). These heat-induced changes in anther development and tapetum degradation may partly explain the high pollen sterility of LYPJ plants (Fig. 6). In contrast, the well-developed anthers of SY63 plants enhanced their heat tolerance in terms of pollen viability (Fig. 6).

### The relationship among anther structures, anther dehiscence and pollen shedding

Pollen reception (pollen numbers on stigma) influences spikelet fertility under HT [1, 13, 29]. Wu et al. [4] also found that HT treatment at the heading stage led to poor pollen shedding in heat-susceptible cultivars. In this study, we observed that HT treatment during panicle initiation had a negative effect on anther dehiscence and the pollen shedding percentage of the anthers in LYPJ; however, SY63 showed stable anther dehiscence and pollen shedding under HT and CK (Fig. 1K and L). Poor pollen shedding may be a disadvantage for successful reproduction under HT [12].

**Fig. 5:**
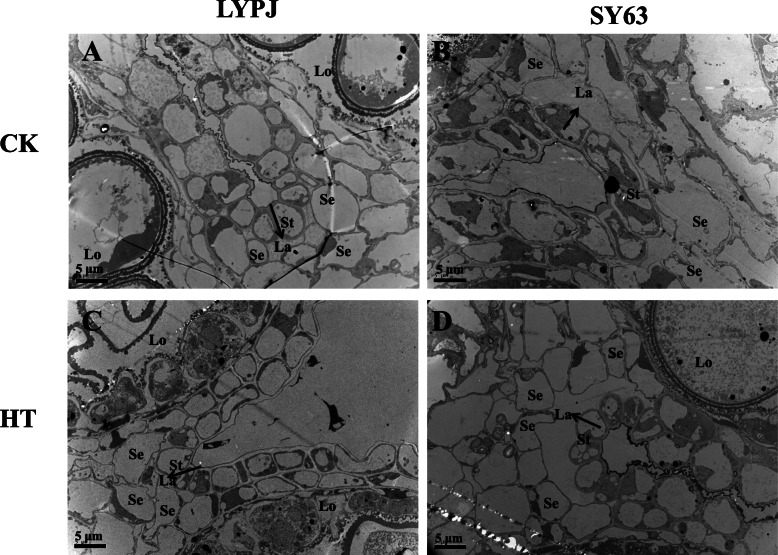
Effects of high temperature treatment on the anther stomium septum and lacuna at stage 10. Arrows label La (lacuna formed between the septum and the stomium); Lo, locule; St, stomium, Se, septum. CK, control temperature; HT, high temperature

**Fig. 6:**
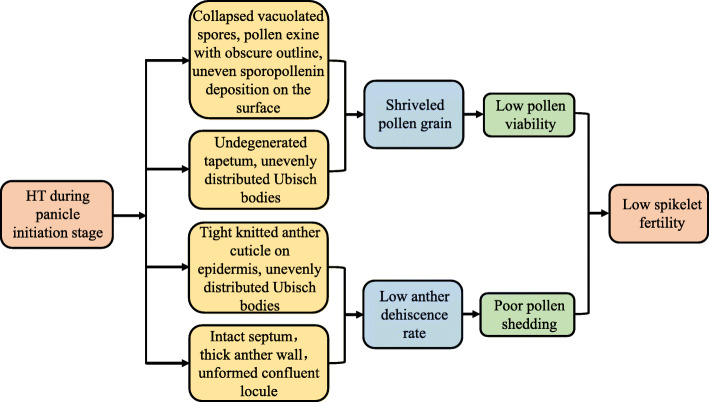
A schematic diagram of the effect high temperature treatment on spikelet fertility. HT, high temperature

The existence of the tapetum at flowering may halt anther dehiscence [26]. Our microscopic observations demonstrated that the tapetum did not degenerate until stage 13 (anther maturity stage) in LYPJ plants under HT (Table 2, Figs. 3C, K, 4O and 5C), and the anther wall (i.e., more remaining cell layers) remained between the locule and the lacuna. Similarly, locules were kept closed by parenchyma and endothecium cells at anthesis due to the remaining anther wall cell layers in rice, which subsequently led to poor pollen shedding under HT [26]. However, cell layers of the anther wall in rice were not affected by HT during panicle initiation, while failure in lysis of the septum cell wall inhibited anther locule opening [27]. In our study, lysis failure of the septum cell wall was also observed in LYPJ under HT at anthesis (Fig. 4O), and the septum cell wall degraded in LYPJ under CK (Fig. 4M), as well as in heat-tolerant SY63 under both CK and HT (Fig. 4N and P). Therefore, our study suggests that both anther wall degradation and septum cell wall lysis together regulate anther dehiscence under HT conditions during panicle initiation. HT treatment during panicle initiation inhibited anther wall degradation and septum cell wall lysis in heat-susceptible LYPJ and subsequently resulted in low anther dehiscence in comparison with that of heat-tolerant genotype SY63 (Fig. 6).

Anther locules are opened via the stomium splitting. In our study, HT treatment did not affect the number of stomium cells in LYPJ or SY63, and the two varieties had similar numbers of stomium cells under CK and HT (Table 2 and Fig. 5). Similarly, Bagha [27] did not find differences in stomium cell abundance between various genotypes grown under CK and HT. Therefore, inhibition of anther dehiscence and pollen shedding at stage 13 under HT may be attributed to septum cell lysis rather than stomium splitting.

We also found that the number of septum cells in SY63 was lower than that of LYPJ under both CK and HT, and HT resulted in a slight increase in the abundance of septum cells in both varieties (Table 2). This result was consistent with the results of Bagha [27], who reported that the number of septum cells was not affected by HT in heat-susceptible or heat-tolerant varieties; moreover, the heat-tolerant variety had fewer septum cells. Therefore, a lower number of septum cells may be a favorable characteristic for anther dehiscence in heat tolerant varieties, and this factor may have contributed to the high heat tolerance of SY63. Our study did not investigate the cause for the cessation of septum splitting in LYPJ under HT. Inhibition of septum cell wall lysis may be attributed to low cell wall invertase activity caused by HT treatment [27]. The underlying physiological mechanism for regulation of septum splitting under HT merits further investigation.

Our study found that HT inhibited pollen shedding of the anthers (Fig. 1G and L). There are two likely reasons for our observation of inhibited shedding under HT. First, Ubisch bodies on the inner surface of the anther wall show non-wettability due to the distribution of hydrophobic substances, and a continuous hydrophobic layer appears on the inner surface due to locule shrinkage after dehiscence [43]. Therefore, the occurrence of Ubisch bodies decreased the sticking properties of the pollen to the locule wall [43, 44], and decreased sticking favors pollen dispersal [43]. HT induced uneven distribution of Ubisch bodies in heat-susceptible LYPJ plants (Fig. 2N), which may be one of the reasons for the reduction in pollen shedding of the anthers (Figs. 1L and 6). Second, anther stomata may play a crucial role in anther dehydration in rice [45], which may favor anther dehiscence and pollen shedding. Recently, in *ICE1* (*ice1–2*) *Arabidopsis* mutants, Wei et al. [46] found that decreased stomata density and abnormal stomata development were not advantageous for anther dehydration and anther water movement, so the epidermis did not shrink to dehisce, regardless of the existence of stomium cells in the anther; these changes resulted in low pollen dehiscence, low pollen viability and a low germination percentage. Therefore, to elucidate the mechanism underlying poor pollen shedding, more investigations, such as studies of anther stomata development and the physiological changes associated with anther dehydration in dehisced anthers, should be performed under HT.

### Possible genetic and physiological causes for high temperature tolerance

Our study and previous report observed the heat susceptibility of LYPJ and heat tolerance of SY63 [3]. Pei’ai64S, the female parent of LYPJ, is thermo-sensitive genic male sterile line, and is sterile when ambient temperature is more than 23.3 °C. Pei’ai64S plants exposed to HT (38 °C) were completely pollen sterile, with different biochemical and physiological responses when compared with plants under 22 °C [47, 48]. The variety 9311, the male parent of LYPJ, showed moderate HT tolerance during anthesis [49, 50]; however, the heat sensitivity of 9311 during panicle initiation is not yet clear. Therefore, it is difficult to determine the parental contributor to the heat susceptibility of LYPJ during panicle initiation. Fu et al. [51] found that male parents had large contribution to heat tolerance of F_1_ hybrid than female parents during anthesis, and Minghui 63, the male parent of SY63, was HT-tolerant during anthesis. Xiong et al. [52] found that Zhenshan 97B was moderate HT tolerant during the whole growth period. Hence, heat tolerance of SY63 may be derived from both parents. Further investigation is needed to explore the genetic relationship between the two hybrids and their parents in terms of heat sensitivity.

In report of Wu et al. [3], heat tolerance of SY63 was partly attributed to the stability of active phytohormones in panicles, such as cytokinins, gibberellin A1, and IAA, the reduction in active phytohormones may be a contributor to heat sensitivity of LYPJ. On the other hand, sugar metabolism may be associated with heat tolerance. Xiong et al. [52] found that compared with the heat susceptible Koshihikari, the HT-tolerant N22 had higher content of nonstructural carbohydrates during the whole growth period under HT; N22 showed higher expression of genes for a sugar transporter (MST8) and a cell wall invertase (INV4), compared with Moroberekan [53]. Thirdly, HT tolerant Tanliangyou83 had higher catalase activity and higher expression of gene *HSP71.1* for heat shock protein in spikelets than the heat susceptible Lingliangyou722 [54]; typically HT-tolerant type rice cultivars had higher activities of superoxide dismutase and catalase in anther than HT-sensitive type cultivars under HT during meiosis [55]. These previous data indicate that heat tolerance in rice is closely associated with the responses of physiological and biochemical processes to heat stresses.

## Conclusions

HT treatment during panicle initiation significantly decreased the grain yield of heat sensitive variety LYPJ by decreasing both the number of spikelets per panicle and the seed setting percentage. The reduction in the seed setting percentage of LYPJ was mainly attributed to lower spikelet fertility, which was caused by decreased pollen viability, reduced anther dehiscence and poor pollen shedding of the anthers. HT had only minor adverse effects on the grain yield of heat-tolerant variety SY63, which was attributed to stable pollen viability, anther dehiscence, and pollen shedding of the anthers. Abnormal anther development and malformation of pollen together resulted in shrunken pollen grains in LYPJ under HT, which may account for poor pollen viability. Abnormal anther epidermis, thickened anther walls, un-degraded septum, inhibition of confluent locule formation and unevenly distributed Ubisch bodies at pollen maturity were partially responsible for the decreased anther dehiscence rate and reduced anther pollen shedding of LYPJ under HT. HT did not substantially change the development of the anther wall and microspores at the late stage of pollen formation in SY63.

## Methods

### Plant materials and growth conditions

A pot experiment was performed during the rice growth season from May to October 2016 at the experimental station of Huazhong Agricultural University, Wuhan, China (30°29′ N, 114°22′ E). Two rice genotypes LYPJ (HT susceptible) and SY63 (HT tolerant) were used in this study. Seeds of two-line hybrid rice LYPJ from the cross of Peiai 64S and 9311 were purchased from Mingtian Seed Co., Ltd., Nanjing city in Jiangsu province, China; Seeds of three-line hybrid rice SY63 from Zhenshan 97A and Minghui 63 were purchased from Chichengsannong Seed Co., Ltd., Suining city in Sichuan province, China. After breaking dormancy at 50 °C in oven for 2 days, seeds of LYPJ were sown in plastic seeding trays with wet paddy soil. Staggered nursery sowing at a 7-day interval (15 May) was performed for SY63 to synchronize the panicle initiation time of LYPJ and SY63. Three four-leaf seedlings were transplanted into a 12 L plastic pot (25.5 cm height × 24.4 cm top diameter) on 5 June, which contained a mixture of 10 kg clay soil with the following properties: pH 6.6, 10.5 g organic matter kg^− 1^, 1.0 g total N kg^− 1^, 8.1 mg Olsen P kg^− 1^, and 113.8 mg exchangeable K kg^− 1^. Phosphate fertilizer (1.50 g P pot^− 1^) in the form of calcium superphosphate and potassium fertilizer (1.50 g K pot^− 1^) in the form of potassium chloride were applied as basal fertilizer. A total of 1.80 g N pot^− 1^ was applied as urea with three splits: 0.72 g N pot^− 1^ as basal fertilizer, 0.36 g N pot^− 1^ top-dressed 12 days after transplanting, and 0.72 g N pot^− 1^ applied as panicle fertilizer. The potted plants were placed outdoors in three replicates, 7 pots for each variety and each temperature treatment per one replicate. All plants were artificially watered with tap water each day, and the surface water layer in the pots was kept in approximately 2 cm during the entire growth season. Additionally, all pots with plants were manually moved every 7 days to avoid positional effects, and fine management was performed during the whole growth season.

### High temperature treatment

Two individual greenhouses (4 m in length, 4 m in width, and 4.5 m in height) were used for temperatures treatment. The control device of temperature and relative humidity and control system in the greenhouses are consistent with the description of Wu et al. [3]

**Fig. 7:**
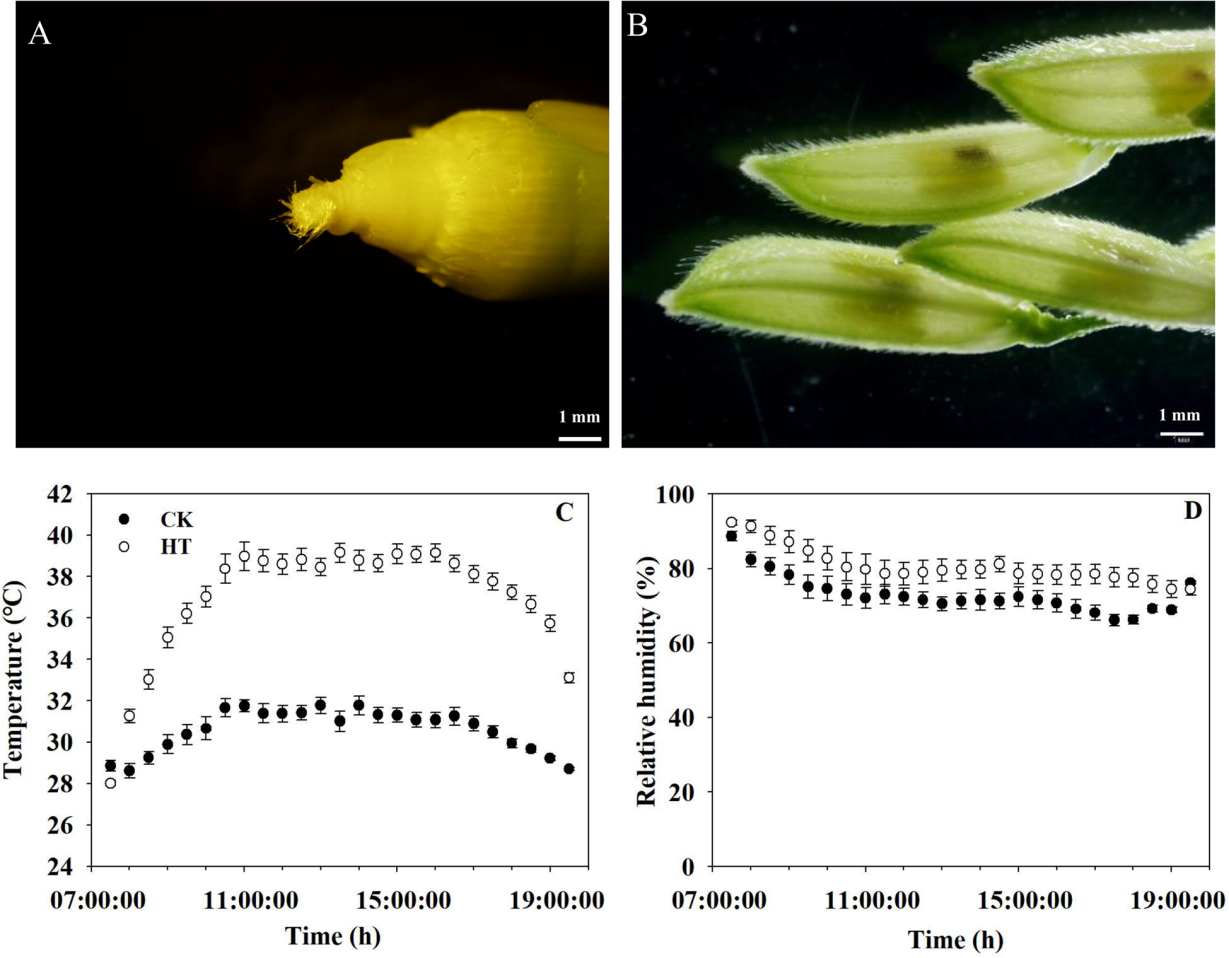
Panicle phenotypes at the beginning and end of temperature treatment and average daytime (7:30–19:30 h) temperature and relative humidity during the entire high temperature treatment period. **A** indicates the young panicle during the 2nd stage of panicle development at the beginning of temperature treatment, **B** indicates the spikelets during pollen maturity at the end of temperature treatment. **C**, **D** are temperature and relative humidity, respectively, data are the average over four sensors ± standard error of the mean (*n* = 4) every half hour (the average over 28 days) during the temperature treatments. CK, control temperature treatment; HT, high temperature treatment

The temperature treatments were performed during panicle initiation. As described by Yoshida [56], panicle initiation stage starts with the neck-node differentiation and ends when the pollen is fully matured, which is often divided into 7 stages; the initiation of panicle primordium starts about 30 days before heading. In this study, high temperature treatment was imposed for 28 consecutive days from the initiation of panicle primordium (the 2nd stage of panicle development, Fig. 7A) to pollen maturity (Fig. 7B).

For the high temperature (HT) treatment, the daytime temperatures were set at 37 °C from 07.30–19.30 h, whereas the daytime temperature was 31 °C for the control temperature (CK) treatment. The nighttime temperature for both temperature treatments was set at 27 °C from 19.30–07.30 h. The daytime relative humidity was set at 80% for both treatments, and the nighttime relative humidity was set at 90%. The HOBO sensors (H08–003-02, Onset Computer Corporation, Bourne, MA, USA) were mounted 5 cm above the canopy for recording temperature and relative humidity [3]. The rice plants were moved to the two greenhouses at the initiation of panicle primordium according to apical dissection and leaf age. Plants were subjected to HT treatment throughout the whole panicle initiation period. The actual daytime average maximum temperature under HT was 39.1 °C, which was 8.0 °C higher than that of the CK treatment. The actual mean daytime temperature during the whole HT duration was 36.9 °C, which was 6.4 °C higher than that of the CK treatment (Fig. 7C). The mean daytime relative humidity of the CK treatment was 73.0% during the entire treatment. The mean daytime relative humidity under HT was 80.6% during the entire treatment (Fig. 7D). The mean nighttime temperature was 27.4 °C under CK and 28.0 °C under HT during the whole HT duration. The mean nighttime relative humidity was 95.6% under CK and 93.7% under HT during the whole HT duration. When the young panicle of the main tiller emerged from the flag leaf sheath, HT treatment was terminated and all plants were moved to natural ambient conditions and grown to maturity.

### Yield and yield components, spikelet fertility, size of spikelet and grain

**Fig. 8:**
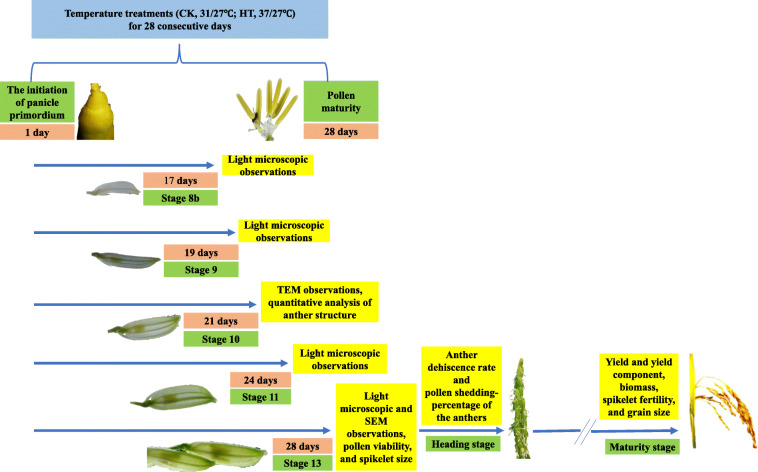
A schematic illustration of sampling and measurements during and after temperature treatment. High temperature (mean daytime/nighttime temperatures, 37/27 °C) and control (31/27 °C) were imposed for 28 consecutive days. During the temperature treatments, spikelets and anther were collected for microscopic observations at 17 days (stage 8b), 19 days (stage 9), 21 days (stage 10), 24 days (stage 11) and 28 days (stage 13), respectively. Pollen viability and spikelet size were analyzed at 28 days. Anther was sampled for measurement of anther dehiscence rate and pollen shedding percentage of the anthers at heading stage; at maturity, plants were collected for determination of yield and yield components, biomass, spikelet fertility and grain size. SEM, scanning electron microscopy; TEM, transmission electron microscopy

General arrangements for samplings and measurements in the study were presented in Fig. 8. At maturity, three plants from three pots were harvested, then all leaves, stems and panicle were collected. After the number of panicles per plant was counted, then all grains were threshed manually. The filled grains were separated from half-filled grains and empty grains by submerging them into tap water. For determination of spikelet sterility, the empty grains were pressed with thumb and forefinger and checked by opening the lemma and palea [1]. Empty grain that had not an embryo was considered as sterile one. The grains of three types were also counted, respectively. Then, all the separated parts (leaves, stems, rachis, the three type grains) were put into an oven at 80 °C for 7 days, then dry weights were obtained. Total biomass (g plant^− 1^) was the total dry weight of the aboveground plants, all filled grains were used to determine grain yield (g plant^− 1^), spikelets per panicle, seed setting percentage (%), 1000-grain weight (g), and spikelet fertility (%) were calculated.

### Pollen viability, anther dehiscence rate, pollen shedding percentage of anthers

More than 150 spikelets were collected from three panicles 1 day prior to anthesis, immediately frozen in liquid nitrogen and stored at − 80 °C (Fig. 8). All indehiscent anthers were excised from 20 non-opening spikelets. Pollen grains were scattered on a slide with 60 μL Alexander solution (Bioshap, China) by manual squeezing using tweezers. Ten minutes later, the Alexander-stained pollen grains were examined at 100× magnification (10 × 10) using an inverted fluorescence microscope (Ti-SR, Nikon, Tokyo, Japan). Pollen stained reddish purple was considered fertile, whereas pollen stained green was considered sterile [57]. The percentage of pollen viability (%) was calculated as the percentage of reddish-purple pollen grains to all pollen grains.

For observations of anther dehiscence and pollen shedding, the opening spikelets of the main panicles were collected from 10.30–11.30 h, then 80–100 anthers were sampled for each replicate. Additionally, six anthers from one spikelet were collected for stereo microscopical observation (SZX16, Olympus, Tokyo, Japan). As descripted by Wu et al. [3], anthers that had opened apical and/or basal pores were considered as dehisced, the anther dehiscence rate (%) was defined as the ratio of dehisced anthers to total anthers.

At the flowering stage, anthers that had completely shed their pollen were white, and anthers on which many pollen grains remained were yellow. The pollen shedding percentage of the anthers (%) was expressed as the percentage of white anthers among all anthers collected. Dehiscent and indehiscent anthers were assessed (Fig. 8).

### Observation of anther structures via light and scanning electron microscopy

Young panicles were selected during the booting stage based on the distance between the collar of the 2nd leaf and the flag leaf collar [58]. According to the divisions of the anther development period [17], the topmost three spikelets of the different panicles were sampled at stage 8b (the distance between the collars of the 2nd leaf and the flag leaf was − 2 cm, indicating that the flag leaf collar inside the sheath of the 2nd leaf was 2 cm below the 2nd leaf collar, spikelet length was 6.0–7.0 mm at this stage) 17 days after HT treatment, stage 9 (the distance was 0 cm, spikelet length was 7.0–7.5 mm) 19 days after HT treatment, stage 11 (+ 7 cm, spikelet length was 8.0-8.5 mm) 24 days after HT treatment, and stage 13 (+ 13 cm, spikelet length was 8.5–9.5 mm) 28 days after HT treatment, respectively; then all anthers of the sampled three spikelets were collected (Fig. 8, Figure S1). The stage of anther development was determined by the length of spikelets [58] (Figure S1). According to spikelet length and the relationship between grain weight and spikelet development processes [59, 60], we roughly estimated the percentage of spikelets that might be at the similar developmental stage. The proportion of spikelets in the same period was 19% under CK and 29% under HT in LYPJ, 20 and 21% in SY63, according to the numbers of differentiated primary branches and spikelets at heading stage in our study (it is assumed that the floret development stage of the topmost five primary branches was same). The collected spikelets were placed in fixation solution (50% ethanol, 5% glacial acetic acid, and 3.7% formaldehyde) for 24 h at room temperature and rinsed with 70% ethanol twice after removing the fixation solution. Fixed anthers were embedded in paraffin after dehydration in an ethanol concentration series (50, 75%, 4 h in each; 85, 90%, 2 h in each; 95%, 1 h; 100%, 30 min), clearing via xylene and paraffin infiltration. The 3 μm thin sections were prepared with a microtome (RM2016, Leica Microsystems, Wetzlar, Germany) and stained with 0.5% toluidine blue solution. Images were captured with an inverted fluorescence microscope (Ti-SR, Nikon, Tokyo, Japan).

Samples for scanning electron microscopy observation were prepared according to the methods described by Min et al. [25] with minor modification. Mature anthers from opening spikelets were sampled (Fig. 8) and pre-fixed in fixation tubes containing 2 mL of 2.5% glutaraldehyde (v/v) in 0.1 M phosphate buffer (pH 7.2) overnight at 4 °C. Next, the anthers were dehydrated in a graded series of ethanol solutions (30, 50, 70%, 10 min in each; 80, 100%, 8 min in each) and immersed three times in isoamyl acetate (10 min in each). The fixed samples were processed via critical point drying using liquid CO_2_ and gold coating, followed by observation using a scanning electron microscope (JSM-6390/LV, JEOL, Tokyo, Japan).

### Observation of the ultrastructure of anthers using transmission electron microscopy

Samples were prepared for transmission electron microscopy observation according to methods described by Cao et al. [61]. At the 10th anther development stage (about 21 days after HT treatment, the collar of the flag leaf was 5 cm above the collar of the 2nd leaf, spikelet length was 7.5–8.0 mm at the stage), 30 spikelets attached to the top five primary branches were sampled from three panicles (Fig. 8), after which approximately 100 anthers were fixed in a fixation tube containing 2 mL of 2.5% glutaraldehyde (v/v) in 0.1 M phosphate buffer (pH 7.2) and vacuumed for 15 min. The tubes containing the anthers were stored at 4 °C overnight. Subsequently, the anthers were washed three times with 0.1 M phosphate buffer (pH 7.2) and post-fixed with 1% (w/v) osmium tetroxide (SPI, SPI Chem, West Chester, PA, United States) for 2 h at room temperature. The samples were washed three times with 0.1 M phosphate buffer at room temperature (30 min each time) and dehydrated via a graded acetone series (30, 40, 50, 60, 70, 80, 90, 100%, 20 min each). The dehydrated samples were infiltrated with gradient acetone-Spurr resin solution (3:1 v/v, 1:1 v/v, 1:3 v/v, Spurr resin, 6 h each), after which each sample was placed into Spurr resin solution, which was polymerized with Spurr resin at 60 °C for 48 h. Ultra-thin sections (90–110 nm) were cut from the polymerized block with a microtome (UC 6, Leica Microsystems, Wetzlar, Germany) and mounted onto copper grids, following by observation using transmission electron microscopy (TEM) (H-7650, Hitachi, Tokyo, Japan) and a CCD camera (Model 832 ORIUS, Gatan, America).

The TEM images were used to quantify the average tapetum area per cell, number of vacuolated microspores per locule, stomium cells per cross-section, septum cells between the bundle sheath and sub-stomial lacuna, and septum cells between the stomium and locule via image analysis software (National Institute of Health, Bethesda, MD, USA). Stomium cells were distinguished according to whether their cell walls had smooth cuticles; non-stomium epidermal cells had crenulate cuticles [27]. In addition, vacuolated microspores, pollen exine, the distribution of Ubisch bodies, and the morphologic structure of the anther walls were also observed and characterized using the TEM images (Fig. 8). Two anthers for each replicate were used for TEM observations, and two slices of each anther were used to characterize anatomical structures.

### Statistics analysis

The value for a given trait was expressed as the mean of three replicates with standard error (SE) using the SigmaPlot 10.0 software package (SPSS Inc., Chicago, IL, USA). Differences between the two temperature treatments for the same variety and between the two cultivars under the same temperature treatment were evaluated using the least significant difference (LSD) test at a 5% probability level using Statistix 9 software package (Analytical software, Tallahassee, FL, USA).

## Supplementary Information

**Additional file 1.**

## Data Availability

All the data on the present study has been included in the tables and/or figures form in this manuscript; and the datasets used and/or analyzed in this study are available from the corresponding author on reasonable request.

## Abbreviations

CK: Control temperature
HT: High temperature
