# Supplementary material for: Abnormal anther development leads to lower spikelet fertility in rice (Oryza sativa L.) under high temperature during the panicle initiation stage
Source: BMC Plant Biol. 2021 Sep 20;21:428. doi: 10.1186/s12870-021-03209-w (PMC8451141; doi:10.1186/s12870-021-03209-w)
Supplement: Supplementary file 1 — Additional file 1. [file 12870_2021_3209_MOESM1_ESM.docx]

**Table S1** Effects of high temperature treatment on spikelet size and grain size in LYPJ and SY63

| Cultivars | Treatment | Spikelet size before heading  (mm) | | Grain size at maturity  (mm) | |
| --- | --- | --- | --- | --- | --- |
|  |  | Length | Width | Length | Width |
| LYPJ | CK | 9.36±0.03 ^a*^ | 3.16±0.05 ^a^ | 9.10±0.03 ^a*^ | 2.79±0.04 ^a^ |
|  | HT | 8.84±0.04 ^b*^ | 2.95±0.03 ^a^ | 8.81±0.01 ^b*^ | 2.72±0.02 ^a^ |
| SY63 | CK | 8.72±0.06 ^a^ | 3.42±0.04 ^a*^ | 8.68±0.02 ^a^ | 3.11±0.02 ^a*^ |
|  | HT | 8.39±0.02 ^b^ | 3.21±0.01 ^b*^ | 8.57±0.03 ^a^ | 2.99±0.01 ^b*^ |

*CK* control temperature; *HT* high temperature; Data are the average over three replicates ± standard error of the mean (n=3). The different superscript lower-case letters indicate significance between the temperature treatments for the same cultivar at *P*<0.05. Asterisk indicates significance between the two cultivars for the same temperature treatment at *P*<0.05.

Approximately 50 full length spikelets one day before heading and 100 grains at maturity were randomly selected for measurement of spikelet and grain size. Length and width of spikelets and grains (mm) were measured as described by Wu et al. [3]

Reference

Wu C, Cui KH, Wang WC, Li Q, Fahad S, Hu QQ, et al. Heat-induced phytohormone changes are associated with disrupted early reproductive development and reduced yield in rice. Sci Rep. 2016; 6:34978.

**Fig. S1.**

**Fig. S1.** Spikelets at different anther developmental stages.

A, B, C and D are spikelets (6.0-7.0 mm in length) at stage 8b ; E, F, G and H are spikelets (7.0-7.5 mm in length) at stage 9; I, J, K and L are spikelets (8.0-8.5 mm in length) at stage 11; M, N, O and P are spikelets (8.5-9.5 mm in length) at stage 13. Q and R are spikelets from the same panicle that represent 5 developmental stages (spikelet length is 1.0-3.0 mm at stage 6, 3.0-5.0 mm at stage 7, 5.0-6.0 mm at stage 8a, 6.0-7.0 mm at stage 8b, and 7.0-7.5 mm at stage 9) in LYPJ and SY63 under CK, respectively. Scale bar = 1 mm.
